# Helicopter Emergency Medical Services in 2017 Kermanshah Earthquake; a Qualitative Study

**Published:** 2019-06-10

**Authors:** Ali Sahebi, Zohreh Ghomian, Mohammad Sarvar

**Affiliations:** 1 Department of Health in Disasters and Emergencies, School of Public Health and Safety, Shahid Beheshti University of Medical Sciences, Tehran, Iran.; 2 Department of Helicopter Emergency Medical Services, Ministry of Health and Medical Education, Tehran, Iran.

**Keywords:** Emergency medical services, emergency medical technicians, transportation facilities, air ambulances, disaster planning, earthquakes

## Abstract

**Introduction:**

Becoming aware of experiences, and lessons learned in challenges can help optimize planning and improve efficiency and effectiveness. The present study aimed to address the challenges of helicopter emergency medical services (HEMS) from the viewpoint of the managers involved in HEMS in Kermanshah earthquake.

**Methods:**

This qualitative research was done using the content analysis method. The data were collected by semi-structured interviews.  The study population consisted of directors who participated in management and transfer of injured people in the earthquake-stricken area of Kermanshah. Sampling was purposeful in the first stage and then by the snowballed method.

**Results:**

In the present study, 479 codes were initially extracted regarding participants' perspectives and experiences and after eliminating duplicates, 53 codes were finalized. After analyzing the data, 4 categories and 12 sub-categories were extracted. In this research, lack of integrated management and process-oriented preparedness were the subjects with the highest number of codes.

**Conclusion:**

According to the findings of this study, it is suggested that comprehensive training programs should be implemented for effective management of the air emergency process during disasters such as earthquakes.

## Introduction:

As a natural hazard, earthquake occurs 16 times a year on average all over the world, which causes a lot of human casualties and economic losses (1). According to the natural disasters recorded in 2014, in Eastern Mediterranean and North Africa (MENA) countries, 16 natural disasters were registered, 3 (19%) of which occurred in Iran. The occurrence of these disasters in Iran led to 38 deaths, 452,580 injured and homeless people, and 92,000 US $ economic damages (2). On Saturday, November 12, 2017, at 21: 48 local time, an earthquake with a magnitude of 7.3 on the Richter scale and 11-kilometers depth occurred in Ezgeleh District, Kermanshah Province. The destruction of 8 cities and 1930 villages, as well as 625 deaths and 15000 injured individuals, resulted from this earthquake. More than 1,000 aftershocks were recorded during the days after the earthquake, the largest of which had a magnitude of 4.7 and occurred on November 13th, 2017 (1, 3, 4). In earthquake relief, victims, especially the critically injured, are transported by air emergency due to its high transport efficiency, and low mortality and disability rates. Since ground emergency medical services (GEMS) are time-consuming, Helicopter Emergency Medical Service (HEMS) plays a critical role in providing timely emergency medical services (EMS) for patients in distant areas by reducing the time of transporting patients with critical situations to hospitals (5-8). HEMS plays an important role in the affected areas through triage, providing medical care, treating injured and transferring them to hospitals, as well as in transportation of equipment, personnel, accident victims and meteorological monitoring (9, 10). Although HEMS is more expensive than GEMS, its use is increasing due to its multiple advantages. In fact, HEMS is an essential component of planning a comprehensive and local response to natural and man-made disasters for evacuating the injured people as soon as possible (11, 12). A comparison of the advantages of GEMS and HEMS showed that HEMS is preferred in special circumstances as it can warrant transportation of the injured to faraway hospitals in the least possible time. HEMS also benefits from experienced technicians who can effectively manage the disaster scene and triage of the injured (13, 14). Considering the destruction of infrastructure, the importance of timely and effective responses, and the role of air emergency in the rapid transfer of injured to medical centers, the present study aimed to address the challenges of helicopter emergency medical services from the viewpoints of the managers involved in the HEMS in Kermanshah earthquake. 

## Methods:

***Study design and Setting***

A qualitative study was done with the content analysis approach (CAA) using the Graneheim and Lundman (2004) method, from December 2017 to May 2018. Using CAA, codes, sub-categories, and categories were extracted the by an inductive process (15). In order to conduct the interviews, high-ranked directors of Kermanshah earthquake management team from Tehran, as well as operational managers from Kermanshah and Ilam provinces who participated in air emergency during Kermanshah earthquake were included. 

***Participants***

In the present study, experts and experienced individuals in relief and air transportation handling of the injured people were interviewed in two navigation and medical groups. The key persons involved in national planning for air emergency in Kermanshah earthquake, members of the medical team and air emergency in Iran, experienced HEMS provers to those injured in Kermanshah earthquake, as well as personnel affiliated to the Ministry of Health and Medical Education, the Red Crescent Society, and finally those who were willing to participate in the study, were included. Members of military organizations were excluded from the study due to the impossibility of interviewing.

The first interview was conducted with the head air emergency manager who served in an air emergency during Kermanshah earthquake. After identifying the initial concepts, other participants were identified and selected. Finally, 6 participants were included in two groups until data saturation was reached.

Data collection 

Sampling was purposeful in the first step and then the snowball method was applied. Data collection was conducted through semi-structured and in-depth interviews with open and general questions (challenges HEMS faced during the western province earthquake). At the beginning of the interview the purpose of the study was explained to the participants. Then, the main research question was asked. Data collection was conducted until reaching data saturation and no new issues were introduced. All the interviews were conducted by phone and in person. The interviews lasted between 30 and 55 minutes (an average of 42 minutes). To resolve ambiguity and increase the clarity of the subject, questions were posed based on the participants' impressions. When the participants felt tired and busy, the interviews were interrupted according to their will, and the next appointments were scheduled. With the permission of all participants, all the interviews were recorded using two digital devices and were then transcribed immediately. The interviews were conducted from December 2017 to May 2018.

***Data analysis***

Data analysis was conducted using Graneheim and Lundman's five-step content analysis method (15, 16). These steps included: First, the entire interview was transcribed. The manuscripts of the interviews were accurately read several times to reach an overview of the content. The manuscripts of the interviews were divided into units of meaning. Then, the manuscript was summarized and initial codes were determined. All the codes were classified in terms of conceptual similarities and differences. Finally, categories and sub-categories were formed. After coding the first interview, a set of codes, categories, and sub-categories were extracted. Data were reported based on a scientific approach to qualitative research.

***Trustworthiness***

The participants were chosen from the navigation and medical teams that were involved in HEMS to increase the credibility of the data. Furthermore, the main challenges of this area were addressed. Also, the participants had a rich experience in air emergency, in particular, during Kermanshah earthquake. After analyzing each interview, the participants were revisited to verify the data provided by them (Member check) if needed, corrections were considered. Parts of the interview manuscript along with their codes and categories were sent to a number of experts in the field of qualitative studies to ensure the accuracy of the data analysis (External check). Parts of the interview manuscript along with related codes and categories were also sent to three experts to review the analysis and express their corrective comments. An agreement rate of 85% was observed between the results (Peer check).

***Ethical Consideration***

Verbal informed consent was received from all participants to observe ethical considerations. Then, detailed descriptions were given on the purpose of the research, the interview method, the confidentiality of the information and the participants’ rights to either enter or refuse to participate in the study. Also, no names were recorded in the interviews. The information confidentiality principle was strictly adhered to in each step of the research.

## Results:

***Quantitative findings***

 In the present study, six managers and pilots of the Ministry of Health and Medical Education and Relief and Rescue organization who were directly involved in air emergency following Kermanshah earthquake were selected for interview. All participants were male with the age range of 30-50 and the average age of 40 years old. 40% of them had a Medical Doctor degree and 60% had a bachelor's degree. The information obtained from the participants was based on their management experiences in air emergency in the 2017 Kermanshah earthquake. 

***Qualitative findings***

 In the present study, 479 codes were initially extracted. Considering participants' perspectives and experiences and after eliminating duplicates, 53 codes were finalized. After analyzing the data, 4 categories and 12 sub-categories were extracted (Tables 1-4). In this research, the topic with most codes was the lack of integrated management and process-oriented preparedness. The sub-categories of this topic were interrupted Comprehensive Communication Process, non- systematic registration of statistics and information, lack of identification and tracking of referred injured patients, lack of systematic and parallel interagency coordination, and low prioritization of preparedness plans. 

According to participants’ views, the most important factor influencing air emergency was the lack of integrated management and process-oriented preparation, which had the highest number of codes.

Qualitative method of content analysis resulted in 4 main categories and 12 sub-categories as follows:

***Lack of comprehensive training program***

This was one of the main categories extracted from the interviews. The inefficiency of the triage process, little attention to teaching safety laws and instructions, and ineffective training were the main sub-categories in this category. The most important sub-category was inefficiency of the triage process. In the interviews, it was repeatedly stated that pre-hospital and in-crisis triages were not well delivered, negating the prioritization of injured for being transported by air emergency. In air emergency, the transportation priority is for critical patients who should be transferred to treatment centers for advanced care as soon as possible. It was stated in the interviews that: *“In Kermanshah earthquake, every helicopter landed from every side like a bird. It was not clear whether or not the injured patient they had taken were candidates for aerial emergency or whether they had been under triage or not. The local hospitals in the earthquake area transferred the injured to the helicopter without triage and without any patient identifications*” (Participant number 3, a 37-year old man, air emergency).

**Table 1:** The first category and its sub-categories of Helicopter Emergency Medical Service (HEMS) challenges in Kermanshah earthquake

**Category**	**Sub-categories**	**Codes**
The lack of comprehensiv training program	Ineffective triage	Lack of training for the navigation team regarding triage of injured people following the earthquake
		The low skill of the medical team on how to conduct triage during an earthquake
		The low skill of hospital staff on how to conduct triage for mass casualty
		Failure to implement air transfer protocols for those injured in earthquake
	Paying little attention to safety rules and instructions	Lack of knowledge among the medical team about rules and protocols of flight and navigation
		Inadequate knowledge of healthcare administrators regarding navigation rules
		Low knowledge of hospital staff regarding the safety rules of air emergency
	Ineffective training	Inadequate education of the medical team regarding navigation protocols
		Inadequate education of the personnel deployed to the disaster area regarding air emergency
		Inadequate skill training of the medical team to perform air emergency
		Inadequate educational content regarding the navigation system for hospital staff
		Lack of joint training of medical and navigation teams regarding the air-transport system
		Lack of educational planning in recognizing the physiological impacts of aerial transportation
		Lack of knowledge on the topography of the earthquake-stricken area among the navigation team

**Table 2:** The second category and its sub-categories of helicopter Helicopter Emergency Medical Service (HEMS) challenges in Kermanshah earthquake

**Category**	**Sub-categories**	**Codes**
Limitations in infrastructure development and a comprehensive program-based process	Contingency development for air relief	Insufficient helicopter pads in urban and intercity distances
		Insufficient standard helicopter nests near the earthquake-stricken area
		Difficulties in providing temporary helicopter pads in the earthquake-stricken zone
		The long distance from the airport to the earthquake-stricken area
	Lack of sustainable financial resources	Delay in direct financial aids to air relief system before the start of operation
		Impossibility of financial payments during operation (PayPal)
		Failure to estimate and predict the costs of air emergency in earthquake and crises
		Delay in the financial settlement of the companies involved in air emergency
		High costs of air emergency services
	Shortage in navigational and medical equipment	Insufficient standard navigation equipment
		Insufficient advanced helicopter equipment
		Inadequate helicopters for transferring the injured patients
		Lack of basic medical equipment on the helicopter
		Shortage of specialized medical equipment for a helicopter
		Lack of standard medical equipment for air transportation
		Improper design of helicopter space for applying as an aerial ambulance

**Table 3:** Third category and its sub-categories of helicopter Helicopter Emergency Medical Service (HEMS) challenges in Kermanshah earthquake

**Category**	**Sub-categories**	**Codes**
Lack of integrated management and process-oriented preparation	Disruption in the Comprehensive Communication Process	Lack of a Telecommunication system between the helicopter and the Medical Operations Control Center (EOC)
		Lack of a Telecommunication system between helicopter and hospital emergency
		Lack of a communication system between the helicopter and ambulance
		Out of date navigation equipment
	Non-systematic registration of statistics and information: lack of identification and tracking of the referred injured patients	Lack of a registration system for those injured in the earthquake
		Absence of a checklist for recording the severity of injuries in victims transferred by air emergency
	Lack of systematic and parallel coordination between domains	Lack of a contract between the air transportation system and the oil company to provide helicopter fuel
		Inconsistency between the navigation and medical teams for optimal transfer of the injured
		Absence of an online database for the aviation team to obtain meteorological conditions of the flight and earthquake-stricken area from the Meteorological Center
		Inconsistency in the timely delivery of injured people from a helicopter to the land emergency
		Inconsistencies between Medical, Military, and the Red Crescent teams in the aerial transportation of the injured
		Lack of a joint organization managing others involved in the aerial transportation of the injured in the earthquake-stricken area
		Lack of a comprehensive and coordinated responsive center between organizations involved in air assistance and aerial transportation
	Low priority of readiness programs	Failure to schedule helicopters recruitments to the earthquake-stricken area as soon as possible
		Lack of planning to prepare a hazard map from the earthquake-stricken area
		Lack of air emergency Preparedness Programs in response to crisis/earthquake
		Failure to conduct joint trainings between organizations involved in air emergency
		Lack of contingency programs for air emergency process in earthquake

**Table 4:** Fourth category and its sub-categories of Helicopter Emergency Medical Service (HEMS) challenges in Kermanshah earthquake

**Category**	**Sub-categories**	**Codes**
Lack of attention to the supply and maintenance of specialized human resources	Lack of programs to identify specialized and skilled workforce	Lack of qualified, specialized, and skilled medical staff for delivering air emergency
		The frequent relocation of trained personnel in relief units
		Lack of high-skilled pilots for flying in different weather conditions during natural disasters
	Not encouraging internal motivational factors	Lack of internal motivations for air emergency forces to provide services during disasters
		Low interest in air relief forces to serve in the earthquake-stricken area

***Limitations in infrastructure development and a comprehensive program-based process***

This category comprised of contingency development for air emergency, lack of sustainable financial resources, and shortage in navigation and medical equipment. The most important sub-category of this category was the lack of navigation and medical equipment. Regarding ​​the navigation system, it was stated that these are not advanced equipment. There was no possibility of flying at night for the helicopters, and the mountainous condition of the area had further disturbed the flights. The helicopters did not have an air emergency design, but they had a multifunctional application. Regarding the medical equipment in the helicopters, it was stated that these were unstandardized and unspecialized for air emergency. In fact, these were used in a ground emergency, which was inappropriately implemented on helicopters. It was stated in the interviews *that “Our medical equipment is sound based, which is not fit for being used in helicopters, instead they should have been visual based” (participant number 5, a 45-year old man, Operational Control Center)* and *“The number of aerial ambulances is limited and they cannot all be called at the same time as their missions would remain on the ground” (participant number 1, a 52-year old man, air relief).*

***Lack of integrated management and process-oriented preparedness***

This category included sub-categories of disruption in the Comprehensive Communication Process, lack of a preparation program, non-systematic registration of statistics and information, not detecting and tracing referred injured patients, and lack of systematic and parallel coordination between areas. The most important sub-category in this category was the lack of systematic coordination between areas, which was assigned with the highest number of codes. It was noted that there was a lack of coordination between the air and ground emergency teams in the earthquake-stricken area. There was also a lack of coordination between the helicopters of the medical, military and the Red Crescent organizations and each received commands from their own organizations. Overall, there was no unified command. It was reiterated in the interviews that *“There was no Emergenyc Operating Center (EOC) or air traffic control and guidance center on the disaster scene” (participant number 1, a 52- year old man, air emergency).*

***Lack of attention to the supply and maintenance of specialized human resources***

The sub-categories of this category included the lack of a systematic program for access to skilled and specialized workforce and the lack of reinforcement of internal motivational factors. It was expressed that the air medical team did not have sufficient knowledge and skills regarding air emergency. In addition, trained personnel had been frequently relocated. Also, there was a shortage of skilled pilots to fly in bad weather conditions. There were implications in the interviews as* “Due to the fact that there has not been such a crisis in these areas over the past few years, the personnel of these centers did not know how to admit and transfer the injured to the helicopters” (participant number 4, a 48-year old man, hospital triage)* and* “But the nurse, flight engineers and pilots were not familiar with patient transfer protocols, they have not been sufficiently involved in the educational courses on how to communicate with medical teams and how to lay patients in helicopter. There is little common understanding between navigation and medical teams on flights and patients” (participant number 1, a 52-year old man, air emergency). *

## Discussion:

The present study was a qualitative research on HEMS during Kermanshah earthquake. According to the findings of the study, the main challenges in HEMS in this earthquake included the lack of comprehensive training program, limitations in infrastructure development and a comprehensive program-based process, lack of integrated management and process-oriented preparedness, and lack of attention to the supply and maintenance of specialized human resources.

The results of other studies also indicated that lack of infrastructure and personnel, people and patients’ safety concerns, inadequate resource management, and concerns over the professional competence of personnel have been among the most important challenges faced by HEMS in Iran (17). These challenges imply the necessity of reviewing HEMS in Iran. Monitoring compliance of practice with national standards and laws can play an effective role in improving the quality of HEMS.

Air emergency staff experience high levels of stress due to the closed space of helicopter, noises, vibrations and electronic communications, which necessitate appropriate training (18). Based on the results of this study, one of the main challenges in providing HEMS in Kermanshah earthquake was the lack of comprehensive training proram including inefficient triage process, lack of attention to safety rules and guidelines, as well as ineffective training. 

Most of the participants pointed to the ineffectiveness of the triage process at the site of the incident. The goal of triage in natural disasters and Mass Casualty Incidents (MCI) is to prioritize the need to receive healthcare in stressful, difficult and complex situations. It has been argued that injured patients with less damage may receive faster healthcare in this condition. In such situations, if the health personnel have inadequate knowledge and skills, the triage process may not be performed in accordance with the standard protocols. Furthermore, the intervention of the people and other staff involved in the relief can lead to impetuosity in triage and neglecting air transport indications. Rapid and non-standard triage is done due to limited skill of the personnel. Therefore, education can be used for enhancing the skills, as well as the knowledge of personnel (i.e. effective training) (19). 

The medical and navigational personnel as well as other paramedics should constitute a single working unit in the process of air transport during natural disasters, especially in those with MCI. Therefore, team training with a comprehensive approach including both common and specialized educational topics is needed. Also, educational programs should be able to enhance medical and operational skills and knowledge. Effectiveness of the education can also be evaluated through drills and exercise programs, which will lead to improvement of the process. 

Another major challenge in air emergency during Kermanshah earthquake was the lack of attention to the supply and maintenance of specialized workforce. The participants noted that in Kermanshah earthquake air emergency team, there was a shortage of skilled and experienced staff and doctors in helicopters. Air emergency is a specialized and effective relief service in disasters and requires expert and experienced personnel due to the different physiological responses of the human body on the ground and in the air. On the other hand, air transportation is carried out for highly injured patients who require complex and careful interventions and therefore, the provision of these services requires a high level of knowledge and skills. Air emergency personnel play an effective role in managing medical responses to major accidents through rapid provision of advanced healthcare services (20). 

Also, the presence of doctors in the helicopters can be useful because they can reduce the casualties by timely medical diagnosis, providing specialized services and treatments on the scene, and finally, injured patients can be properly transferred to specialized centers (21, 22).

It was stated that insufficient attention was paid to teaching safety rules and regulations prior to Kermanshah earthquake air emergency. Lack of knowledge and not being aware of these rules and regulations can be hazardous for both medical and navigation teams. Actually, air emergency teams usually pay less attention to the standard flight rules and regulations during the disasters with vastly destructed infrastructure and continuous increase in mortalities and injuries; and instead, focus on the transfer of injured patients to healthcare centers as soon as possible. This situation can endanger both the patient and the rescue team. Simultaneous training of navigation and medical teams improves the safety of rescuers and leads to positive outcomes in recovering patients. This can also augment effective management of difficult scenes and complications by the rescuer teams (23). 

Another major challenge in this study was the lack of integrated management and process-oriented preparedness, which received the most codes. In fact, one of the sub-categories repeatedly expressed by the participants was the lack of a systematic approach to patient care between areas and parallel coordination. The process of air transportation is a complex operation performed by the participation of various organizations. These organizations, however, have their own rules and guidelines. Therefore, the implementation of specific rules and procedures of these organizations in response to disasters results in incoordination. According to the participants, in Kermanshah earthquake, the Medical Operation Control Center did not monitor helicopters, and they transported the patients in coordination with their own Operational Control Center (EOC). In air emergency, using online system and satellites, the incident area is controlled by managers of various organizations involved in disasters. This coordinated process increases the knowledge and awareness of the managers regarding the situation (24). The command and control system is used to create coordination at the scene of the incident, and the dispatch is used to establish coordination among different relief units (25). The implementation of a unified and integrated command system in the site of an incident can enhance organizational coordination. The incident command system (ICS) is an equally structured management tool for coordinating and controlling respondent teams in major accidents. The existence of structures such as the EOC and ICS can be of help in response to disasters (26, 27). 

Limitations in infrastructure and process development based on a comprehensive program have been among other challenges in air emergency during Kermanshah earthquake. Shortage in sustainable financial resources was one of the subcategories in this category. A lot of money is spent on the maintenance of helicopters, pilot training, and each air emergency mission. Given the high costs of air emergency services, it is essential that their missions be equipped with appropriate triage, as well as standards and protocols for air transportation of patients. This will result in proper application and cost-effectiveness in this process. In other countries, the cost of each mission and every hour of the mission of the air emergency is high and reaches several thousand dollars. The difference in the costs of air emergency is due to the types of recruited helicopters, rescue staff, and the duration of the mission (28).

**Recommendations:**

The following recommendations are made for improving the quality of air emergency transportation services in emergencies and disasters:

1. Establishment of a central coordinating center for unified management of Air emergency transportation between the military, ministry of healthcare, and Red Crescent organizations. 

2. Integrated education and training courses for all team members involved in the process of patient delivery in air emergency transportation services during flight based on assessment of educational needs.

3. Using modern medical helicopters that have been equipped with advanced medical and flight navigation equipment.

***Limitations:***

In this study, access to and interview with military experts were not possible; however, if access would have been possible, researchers could benefit from the experience of military experts in Helicopter Emergency Medical Services. 

## Conclusion:

According to the findings of this study, it is suggested that comprehensive training programs should be implemented for effective management of air emergency process during disasters such as earthquakes. 
